# Mental health among children seeking asylum in Denmark – the effect of length of stay and number of relocations: a cross-sectional study

**DOI:** 10.1186/1471-2458-8-293

**Published:** 2008-08-19

**Authors:** Signe S Nielsen, Marie Norredam, Karen L Christiansen, Carsten Obel, Jørgen Hilden, Allan Krasnik

**Affiliations:** 1 Department of Health Services Research, Institute of Public Health, University of Copenhagen, Øster Farimagsgade 5A, DK-1014 Copenhagen K, Denmark; 2 Danish Red Cross Asylum Department, Sandholmgaardsvej 40, 3460 Birkerød, Denmark; 3 Department of General Medicine, Institute of Public Health, University of Aarhus, Building 1260, Vennelyst Boulevard 6, 8000 Århus C, Denmark; 4 Department of Biostatistics, Institute of Public Health, University of Copenhagen, Øster Farimagsgade 5B, DK-1014 Copenhagen K, Denmark

## Abstract

**Background:**

The process of seeking asylum and the related organisational conditions in the host country may adversely affect the children's mental health. The objective of this study was to examine the mental health of children seeking asylum in relation to organisational factors of the asylum system including length of stay and number of relocations.

**Methods:**

The population included all 260 parent-accompanied asylum-seeking children aged 4–16 years living in the asylum centres managed by the Danish Red Cross in October–December 2006. Mental health was evaluated using the Strengths and Difficulties Questionnaire. School teachers evaluated children aged 4–16; and the 11–16-year-olds completed the self-report version. To assess the association between organisational factors and mental health, binary logistic regression analyses were done using backwards elimination. We received responses for 246 children equivalent to 95% of the study population.

**Results:**

Using teachers' reports, we found that children who had been asylum-seeking for more than one year in Denmark had an increased risk of having mental difficulties (odds ratio 5.5, 95% CI 1.8–16.3); four or more relocations in the asylum system were also associated with a higher risk (3.0, 1.4–6.7). When the self-report data were included, the associations were even stronger.

**Conclusion:**

Protracted stays at asylum centres and multiple relocations within the asylum system appear to have an adverse effect on asylum-seeking children's mental health. A limit to the duration of the children's stay in the asylum system should be ensured. Follow-up studies with inclusion of other conditions, such as parental mental health and the children's previous trauma, are needed to clarify the influence of the different factors and their interactions.

## Background

Children seeking asylum not only suffer mentally from conflict-related exposures before migration, during the process of seeking asylum the organisational conditions in the host country may also adversely affect their mental health [1-3]. The literature shows several environmental risk factors for mental illness in refugee children, such as number of transitions, time taken for immigration status to be determined, time spent in the host country, and cultural isolation [1,2,4,5]. In particular, prolonged stay within the asylum system, including detention, has shown to have an adverse mental health and psychosocial effect on both adult and child asylum seekers [3,6-10]. Focussing on the children, by retrospective comparisons, Steel et al. found that asylum-seeking children displayed a tenfold increase in psychiatric disorders subsequent to detention [3]. Mares et al. had similar findings of very high levels of psychopathology in asylum-seeking children, of which much was attributable to traumatic experiences in detention [11]. Nonetheless, both studies were limited by small sample sizes of 20 children, and Mares et al. used a non-standardised diagnostic tool and were restricted to a sample referred for clinical care; however, the strengths lie in the clinical assessment used in both studies. To date, data are scanty and to our knowledge the influence of different organisational factors of the asylum system on asylum seekers' mental health has been estimated only among adults [7-9,12,13].

During children's stay at asylum centres they may experience isolation, crowding, sensory overload, and language problems [3,14]. Furthermore, a protracted stays at asylum centres may lead to a feeling of loss of control and meaningfulness, family instability, and mental illness in parents. This, combined with the general uncertainty and stress of being a refugee and asylum seeker along with disturbing experiences in the past, all contribute to the development of child mental illness [1,3,7,8,10,13-15]. While offering prevalences as different as 7% and 93%, dependent on sample and assessment tools, the international literature makes it clear that psychopathology of various degrees of severity among children seeking asylum is frequent [1,2,4,16,17].

The conditions offered to asylum seekers vary worldwide, influenced by a range of factors including number of arrivals, socioeconomic factors in the recipient country, the degree of sophistication of the prevailing asylum system and the political climate [6,18]. In Denmark, asylum seekers are accommodated at one of the country's eight asylum centres; six centres are run by the Danish Red Cross and two by local authorities [19]. The asylum seekers receive a cash allowance from the Immigration Service to cover their expenses. Asylum seekers may not work in Denmark unless they have a residence and work permit [19]. The majority of the children attend day care or school managed by the Danish Red Cross and a few attend municipal or private primary and lower secondary school; the teaching is in Danish. The children are offered a number of leisure activities as well as being offered the possibility of participating in municipal activities [14,19]. In terms of comparable figures, the duration of the typical applicant's stay tripled from 2001 to 2005 (313 vs. 927 days on average) [20]. Among children seeking asylum in Denmark, an increasing number are referred to psychiatric investigation and treatment, just as the municipalities handle a far greater number of children who require arrangements on the basis of the Law of Social Service [20]. These conditions have led to concern regarding the children's mental health; however, it is unknown whether a relation exists between the organisational factors within the Danish asylum system and the children's mental health.

As this research area lies in the intersection between politics, human rights, ethics, law, and medical science it is highly controversial [21] and is at risk of being intermixed and distorted by both society and asylum seekers. Still, there is a compelling need to develop the evidence-base in this field. The objectives of the present study were to investigate whether mental health among children seeking asylum in Denmark is affected by length of stay and number of relocations within the asylum system.

## Methods

### Population

The population consisted of parent-accompanied asylum-seeking children 4–16 years of age living in the asylum centres managed by the Danish Red Cross at the time of data collection, October–December, 2006. Of a total of 260 children, responses were obtained for 246 individuals (95%): 239 teacher-responded questionnaires (92%) supplemented by self-reports from 88 of the 11–16-year-olds (79%).

### Outcome measures

To assess asylum-seeking children's mental health, the extended version of the Strengths and Difficulties Questionnaire (SDQ) was used within a cross-sectional study design. The questionnaire is available at a web address [22] in 64 languages. It is a screening instrument for child psychiatric disorder which can detect children with psychiatric disorders with reasonable efficiency in community samples [23] as well as in vulnerable groups [24]. Furthermore, the SDQ has been successfully used in both developed and developing countries [25], which provides evidence for the cross-cultural robustness of the questionnaire. The respondents can be parents and teachers of 4–16-year-old children as well as 11–16-year-old children themselves. It is found that a combination of responses from different respondents improves the prediction of psychiatric disorder of the questionnaire [23].

The SDQ consists of 25 assertions about the child's behaviour and it can be allocated to 5 subscales with 5 items each: emotional symptoms, hyperactivity, conduct problems, peer-problems, and pro-social behaviour [22]. The Total Difficulties Score can be calculated by aggregating 20 of the assertions which can each be scored 0, 1, or 2 points in accordance with the child's behaviour. The SDQ also contains an impact supplement that asks about the burden of problems as perceived by the respondent, and the Impact Score is estimated on the basis of the degree of severity of the burdens [26]. As a measure of the asylum-seeking children's mental health, we used the overall SDQ-prediction of mental difficulties, which is calculated by a computerised algorithm based on a combination of the subscales: emotional symptoms, hyperactivity, and conduct problems as well as the Impact Score from multi-respondents. The technical details of the algorithm used are specified in [27]. The SDQ-measure can be seen as an indicator of psychopathology warranting an ICD-10 psychiatric diagnosis [22,23]. Depending on the generated mental difficulties-sum, the children are conventionally placed in three categories: psychopathology 'unlikely,' 'possible' and 'probable'(cut-offs, 80 and 90 percentiles) [22,23] which complies with the official SDQ manual [22,23]. The SDQ and its conventional interpretation are detailed in [22,26].

### Data collection

Teachers of the 4–16-year-old children and the 11–16-year-old children themselves were included as respondents. The responding teachers had substantial contact with each of the children to whom they were giving scores. Parents were not chosen as respondents due to logistic problems and funding limitations.

With the parents' written permission, given after personal information to each family by the first author (SSN) to secure that the parents understood the aim of the study, the 11–16-year-olds filled in the SDQ singly in a separate room at the school. The author (SSN) was present in the room to inform the children about the investigation and to assist any child who had difficulties in understanding. The children could choose to answer the SDQ in Danish or in their mother tongue. The vast majority chose to answer the SDQ in Danish, and only eight children who had recently arrived needed, and got, an interpreter. The children who were not at school on the day of data collection and those who attended a community school were given the questionnaire during a home visit.

For the 23 of the 11–16-year-olds not taking part in the study the reasons were: untraceable, moved within or departed from the country (N = 10), parents' refusal (N = 5), rarely at home at the asylum centres (N = 5), and children's own refusal (N = 4).

Subsequently, data were linked to selected background variables which were: sex, age, nationality, family size, number of parents in Denmark, length of stay in the asylum centres, number of relocations within the asylum system, and administrative phase of the asylum application (1 = unravelling of asylum case, 2 = asylum case work and 3 = asylum denied. Yet, phase 3 also comprises asylum seekers who have obtained asylum and are awaiting placement to a municipality) [20]. These variables were found by searching the internal asylum database of the Danish Red Cross as well as the Immigrant Information Portal of the Danish Immigration Service.

### Statistical methods

Based on the humanitarian announcements from the Danish Red Cross advocating a maximum of one year of stay within the asylum system [28], and political announcements from the Minister of Refugee, Immigration and Integration Affairs regarding a maximum of three relocations within the asylum system [29], we categorized length of stay as 0–12 months versus 13+, and number of relocations as 0–3 versus 4+ relocations. Supplementary analyses were based on the actual covariates as a check but did not provide added insight (not shown). Regarding the overall outcome measure, the categories psychopathology *unlikely *and *possible *were taken as one category versus psychopathology *probable*. To assess the association between organisational factors and mental health, binary logistic regression analyses were carried out using backwards elimination. The analyses were performed in SPSS version 15.0.

### Ethical considerations

Under Danish law, only investigations which include human biological material must be reported to and approved by the Danish National Committee on Biomedical Research Ethics; this does not apply to questionnaire surveys, interview studies and register research surveys [30]. This study required permission from the Danish Data Protection Agency only [31]. This permission was granted. Families received a written introduction to the study comprising a general description of the study including the aims and methods. It was underlined that the children were anonymous and that the investigation would have no influence on the children's asylum case. The letter was translated into the mother tongues of the asylum seekers, as were the consent forms signed by parents. For the analysis, an anonymised database was created.

## Results

Background characteristics of the study population are shown in table 1. Boys were slightly in the majority (58%); 57% were 4–10-year-olds. Children from former Yugoslavia made up 48% of the study population, children from Iraq 27%, and children from all other countries constituted the last 25%. The majority were accompanied by both parents and several siblings. Eighty-six per cent of the children had been asylum-seeking for more than one year, and the average duration of stay was four years. The average number of relocations in Denmark was almost six asylum centres for each child. Two thirds of the children were in administrative phase 3 of the asylum application.

**Table 1:** Background characteristics of the study population

		**Number**	**%**	**Average**
**Total number**		246	-	
**Sex**	Girl	104	42	
	Boy	142	58	
**Age**	4–10-year-olds	139	56	9.6 years
	11–16 year-olds	107	44	
**Nationality**	Former Yugoslavia*	118	48	
	Iraq	67	27	
	All other countries**	61	25	
**Family size**	1–3 members	32	13	4.7 members
	4–8 members	214	87	
**Number of parents**	Single parent	44	18	
	Both parents	202	82	
**School**	Centre school	175	71	
	Community school	23	9	
	Nursery	48	20	
**Length of stay**	1–12 months	35	14	48.4 months
	13–91 months	211	86	
**Number of relocations**	0–3 number of relocations	47	19	5.6 relocations
	4–13 number of relocations	199	81	
Administrative phase of asylum application***	Phase 1 and 2	91	37	
	Phase 3	154	63	

* Albania (4), Bosnia-Herzegovina (10), Yugoslavia (18), Kosovo (66), Macedonia (9), Serbia/Montenegro (10), Slovenia (1).

**Afghanistan (1), Armenia (5), Azerbajdjan (1), Iran (11), Kazakhstan (1), Libya (1), Lithuania (1), Pakistan (2), Russia (8), Somalia (12), Sri Lanka (1), The state of Palestine (9), Stateless (5), Syria (2), Ukraine (1).

*** Phase 1: From arrival until decision is taken whether the application can be under active consideration in Denmark according to the Dublin procedure. Phase 2: From the start of the active consideration of the asylum case until a residence permit or a final refusal of residence is given. Phase 3: After a final refusal of residence and humanitarian residence permit is given. This phase also comprises cases which are en route between the authorities as well as asylum seekers who are awaiting placement to a municipality [20].

Regarding Total Difficulties Score, the teacher-reports indicated that 31% of the children had mental difficulties (table 2). Similarly, the children's own reports showed that 26% had mental difficulties. The teachers' assessment of the number of children with mental difficulties was equivalent to the Impact Score indicating that 31% were perceived as being burdened by their problems. No difference in these outcome measures was found between the older and the younger age groups based on teacher-reports. Nevertheless, the older children had a tendency to show more emotional problems, whereas the younger children showed more hyperactivity problems as well as poor pro-social strengths. Focussing on the self-reports, half of the 11–16-year-olds reported emotional problems. Compared to the responses from the teachers more among the 11–16-year-olds also reported a high Impact Score.

**Table 2:** Frequency table of mental health* among children seeking asylum

		**Teacher-reports of the 4–16-year-olds**	**Teacher-reports of the 4–10-year-olds**	**Teacher-reports of the 11–16-year-olds**	**Self-reports of the 11–16-year-olds**
		**N = 246**	**N = 139**	**N = 107**	**N = 88**
		**%**	**%**	**%**	**%**
**Total difficulties Score**	Normal	51	53	49	37
	Borderline**	14	14	14	19
	Abnormal	31	31	31	26
	Missing	4	2	6	18
**Emotional Symptoms Score**	Normal	67	72	61	24
	Borderline**	10	10	9	8
	Abnormal	20	18	24	50
	Missing	3	1	6	18
**Hyperactivity Score**	Normal	62	60	65	55
	Borderline**	8	8	7	9
	Abnormal	27	31	22	18
	Missing	3	1	6	18
**Conduct Problems Score**	Normal	62	61	64	55
	Borderline**	9	12	6	16
	Abnormal	25	25	24	11
	Missing	4	2	6	18
**Peer Problem Score**	Normal	66	65	66	45
	Borderline**	7	8	5	19
	Abnormal	24	26	23	19
	Missing	3	1	6	17
**Pro-social Behaviour Score**	Normal	65	63	68	77
	Borderline**	8	9	7	3
	Abnormal	22	25	16	3
	Missing	5	3	8	17
**Impact Scores**	Normal	50	53	47	22
	Borderline**	15	14	16	9
	Abnormal	31	30	31	50
	Missing	4	3	6	19

* The categorisation is formed on the basis of a manual based on a British background population.

** Out of these borderline cases, former investigations of the prediction of SDQ have shown that around 10–26% have a psychiatric diagnosis and about 74–90% do not have a psychiatric diagnosis [23,24].

On the basis of the teachers' responses, 35% of the total group of children showed evidence of having mental difficulties (table 3). A significant sex difference was found with boys having worse overall mental health compared with girls – mainly in the area of conduct problems, hyperactivity, and poor pro-social behaviour (not shown in tables).

**Table 3:** Frequency table of psychopathology* unlikely, possible and probable estimated on the basis of the teachers' responses and a combination of the teachers' and the children's responses, respectively

**Psychopathology**	**4–16-year-old children**		**11–16-year-old children**	
	**(Teachers' responses)**		**(Combination of teachers' and the 11–16-year-old chi ldren's responses)**	
	**N = 246**	**%**	**N = 107**	**%**
Unlikely	118	48	29	27
Possible**	42	17	16	15
Probable	86	35	62	58

* The categorisation is formed on the basis of a manual based on British data (background population and clinical child psychiatric material).

** Former investigations of the prediction of the SDQ have shown that of these children with an estimated psychopathology possible around 10–26% have a psychiatric diagnosis and around 74–90% do not have a psychiatric diagnosis [23,24].

Focussing on the 11–16-year-olds, we combined their teachers' responses with the children's own (table 3). Thereby, 58% showed evidence of having mental difficulties. Concerning the 11–16-year-olds' self-reports, a sex difference was found on emotional problems (girls) and behavioural problems (boys) but no sex difference was found in the overall outcome measure of mental difficulties.

In the total material, we found an association between length of stay and mental difficulties (table 4). Based on the teachers' responses, children who had been asylum-seeking in Denmark for more than a year had a marked increased risk (OR = 5.5) of having mental difficulties. No difference in the outcome measure was found between the 4–10-year-olds and the 11–16-year-olds.

**Table 4:** Logistic regression analyses of the adjusted association between the selected risk factors and the outcome measure psychopathology *probable*. The OR is adjusted for the effect of sex

**Risk factors**	**Psychopathology probable**		**Adjusted OR**	**(95% CI)**	P*
	**(n/N)**	**(%)**			
**4–16-year-old children (a)**					

**Length of stay**					
0–12 months	(4/35)	11.4	1.0		
13–91 months	(82/211)	38.9	5.5	(1.8–16.3)	0.002
**Number of relocations**					
0–3 relocations	(9/47)	19.1	1.0		
4–13 relocations	(77/199)	38.7	3.0	(1.4 – 6.7)	0.006

**11–16-year-old children (b)**					

**Length of stay**					
0–12 months	(1/16)	6.3	1.0		
13–91 months	(61/91)	67.0	30	(3.8 – 237)	0.001
**Number of relocations**					
0–3 relocations	(4/18)	22.2	1.0		
4–13 relocations	(58/89)	65.2	6.7	(2.0 – 22.2)	0.002

(a) Based on teacher-responded SDQs for the 4–16-year-old children.

(b) Based on teacher-responded SDQs for the 11–16-year-old children and the self-reported SDQs for the 11–16-year-olds.

* A Wald significance-test at a 5% level was used.

Focussing on the 11–16-year-olds, a combination of their teachers' responses and the children's own (table 4) showed an even stronger association, as the children who had been asylum-seeking in Denmark for more than one year had a considerable increased risk (OR = 30) of having mental difficulties.

Number of relocations was also associated with mental difficulties in the total material as the children who have stayed in four or more different places had an odds ratio of 3.0 of having mental difficulties. A combination of the teachers' responses and the children's own revealed that the odds ratio of mental difficulties among children aged 11–16 years who have stayed in four or more different places was 6.7. In these regression analyses, only sex as confounder remained significant.

The number of relocations evidently increases with the time spent in centres, so the illness patterns of table 4 may reflect the same series of stressful exposures. In fact, once duration of stay was taken into account, the number of residences was no longer significantly predictive, and vice versa. Thus, both factors must be close reflections of the true causal structures underlying the mental health problems we have studied.

## Discussion

The investigation strongly suggests that children seeking asylum develop psychiatric symptoms as a consequence of protracted stay at asylum centres and multiple relocations. These effects on asylum-seeking children's mental health have not been estimated before, but the results confirm those of previous studies of the association between post-migration environmental stressors and asylum-seeking children's mental health [1,3,11]. Most likely a complex interplay between several factors explains our results, and their unique importance is still not fully known.

Furthermore, the children have notably worse mental health in comparison to a European background population where the proportion of children with mental difficulties is approximately 10% [22,32]. Similar differences have been found in former studies with indigenous European children compared to asylum and refugee children in which SDQ was used [33,34].

### Strengths and limitations of the study design

The strengths of this study lie in the relatively high number of study subjects and the low non-response rate, which is rare when dealing with this vulnerable and fluctuating population. Additionally, the use of a validated and widely used screening instrument must be considered a strength. The most important limitations of the present study include the cross-sectional design and the fact that the children were not subject to a clinical investigation.

As the current study had a cross-sectional design, the observed associations between the included variables are not necessarily causal. Nonetheless, there is overwhelming evidence that the process of seeking asylum is both directly and indirectly a stressful and disturbing experience [3,7-9,12,15,35], and the longer the time spent within the asylum system the higher the risk of developing mental disorder among adults [7-9,13]. Although, former studies on this subject have been carried out among a small number of children, they still show similar findings [3,11]. In addition, evidence exists that families' stressful experiences and parental mental health – also during post-migration – have a harmful influence on the children [1,3,14,36]. Thus, the causal interpretation of an effect of the length of stay at the asylum centres on the children's mental health is likely; however, the present estimated odds ratio values must be interpreted cautiously.

In quantifying the outcome measure of mental difficulties, there is a possibility of it being influenced by a number of uncertainties. Firstly, the children were not subject to an individual psychiatric or psychological investigation. In order to assess validity of our screening, it would have been relevant to let the children with high scores undergo an individual psychiatric investigation but because of anonymity that was not possible. Conversely, the SDQ is an internationally validated screening tool with a sensitivity of 63–85% and a specificity of 80–95% as well as positive and negative predictive values of 53–74% and 89–96%, respectively [23,24]. Yet, there are several ways to handle the SDQ-scores and the way chosen makes some difference regarding the estimated outcomes [37]. On the background of the present study, we cannot estimate the occurrence of psychiatric disorder with certainty for which reason the strength of the association between the organisational factors and the mental health might be somewhat imprecise.

Secondly, the assistance of a researcher during the children's responses may have influenced the children's replies towards both under- and overrating of symptoms due to, for instance, reluctance to expose his or her own vulnerability or an interest in gaining sympathy. Thirdly, linguistic misunderstandings may have occurred in the children's replies to the questionnaires. However, this problem seems significantly reduced by the fact that the researcher took steps to ensure that the children understood each single question and answer category by dialogue and exemplification as well as employment of an interpreter in special cases. Furthermore, the problem was reduced by the use of different data sources. Nevertheless, the 11–16-year-old children reported a considerably higher number of symptoms relative to the teachers' replies. The children's high self-report could reflect an actual overrating arising from a wish to strengthen own asylum case by appearing mentally affected; however, it could also reflect that the 11–16-year-olds had severe mental problems that were far more common than their teachers realised. The same pattern has been seen in former investigations of children in the general population [38]. Potential language barriers limiting the teachers' observations were unlikely as the teachers completed the SDQs only for children who they knew well, implying that the children spoke reasonable Danish and had not arrived very recently. Fourthly, the use of two respondents in itself implies casting a wider net. At least with the standard cut-off scores used here, the result is that more children were seen, rightly or wrongly, as having mental difficulties. Hence, when combining teachers' and children's responses we may have overestimated the true prevalence of mental ill-health.

### Factors not investigated

For a large part of the children, the measure of length of stay in the Danish asylum system represents a minimum time of risk since neither time spent during the journey to Denmark was recorded nor the time spent outside the formal asylum system. This would be the case if the child had gone into hiding for some periods or had been applying for asylum in other countries. Number of relocations embodies a minimum estimate for the same reasons. These periods have an impact on the absolute exposure of traumatic events, which can be a contributing factor to the high level of mental illness found among the children [1,5]. The high level of mental illness is not necessarily a result of the duration of stay in Denmark, but the differences found in this study between the children who had been asylum-seeking one year or less and one year or more, respectively, are presumably true.

It is important to consider whether there might be any differences between the two groups of children (those who have stayed within the asylum system relatively briefly vs. longer) which could explain the differences in their mental health. This matter seems implausible as 1) country of origin was not found to be a significant confounder, 2) the children in question predominantly came from war-torn countries, both those who had been asylum-seeking for years (for instance children from former Yugoslavia) and those who had recently arrived (for instance from Chechnya), 3) the adjacent assumption that resourceful families who are less traumatized are more likely not to continue the asylum process is counteracted by the criteria for obtaining asylum in Denmark which are based on the UN Refugee Convention as well as other international conventions to which Denmark must acceded [19].

Although several factors related to conditions in Denmark were assessed in this study, many other factors were not addressed, such as parental mental health and number of traumatic experiences. Other studies have shown that refugee and asylum-seeking children develop mental illness in response to their parents being functionally impaired because of depression, anxiety, PTSD, or other mental problems relating to the stresses due to the asylum process [1,3,14,36]. Thus, these unexplored variables may have helped to explain some of the associations found.

### Generalizability

The overall response rate was 95%. Beyond that, a drop-out analysis showed no deviation of the various background variables between those participating and those not participating. The study population must therefore be assumed to be representative of the present children seeking asylum in Denmark. The existing asylum population in Denmark is characterised by long duration of stay and many relocations at the asylum centres.

### Implication of asylum politics

Long duration of stay at asylum centres seems to have an adverse effect on the children's mental health. Even though some of the children might be traumatised when they arrive in Denmark, it appears that the time of stay in the asylum system may harm their mental health even more. These findings have implications for both the national and international asylum politics and they underline the importance of scientific research to support observations of health professionals and social caretakers within this field [6,11]. To meet our ethical responsibility, we need to voice the words of the unheard by documenting the consequences of the current asylum politics on child mental health. Former studies suggest that a combination of parental, child, and environmental factors constitute the risk factors of child mental illness [1]. As the recipient countries have control of what conditions they offer children seeking asylum, they should seek to minimise the environmental risk factors. Children seeking asylum are among the most vulnerable in our societies, and it is critical that the asylum systems in Western host countries seek to protect children in accordance with the Convention of the Rights of the Child and other international rights documents.

## Conclusion

Protracted quartering at asylum centres and much relocation within the asylum system seem to have a significant, adverse effect on the children's mental health. Therefore, it is relevant to ensure a limit to the stay at the asylum system. The findings of the present study outline important implications for asylum politics and for the mental health care of children seeking asylum. Follow-up studies with inclusion of other conditions, such as parental mental health and the children's previous trauma, could help us understand the effect and interaction of stressors as well as protective factors during the time in the asylum system – and, above all, to clarify the long-term consequences of the asylum-seeking children's poor mental health.

## Competing interests

Co-author Karen Louise Christiansen is employed at the Danish Red Cross Asylum Department. All other authors declare that they have no competing interests.

## Authors' contributions

SSN conceived the study, participated in its design and coordination, collected the data, performed the statistical analyses and interpretation of data and drafted the article. MN conceived the study, participated in its design and interpretation of data and revised the article. KLC conceived the study, participated in its design and coordination and in the interpretation of data, and revised the article. CO participated in the study design and interpretation of data and revised the article. JH participated in carrying out the statistical analyses and in data interpretation, and revised the article. AK conceived the study, participated in its design and interpretation of data and revised the article. All authors read and approved the final manuscript.

## Pre-publication history

The pre-publication history for this paper can be accessed here:


